# Mapping the peer-reviewed literature on accommodating nurses’ return to work after leaves of absence for mental health issues: a scoping review

**DOI:** 10.1186/s12960-020-00478-8

**Published:** 2020-05-19

**Authors:** Christine L. Covell, Shamel Rolle Sands, Kenchera Ingraham, Melanie Lavoie-Tremblay, Sheri L. Price, Carol Reichert, Ivy L. Bourgeault

**Affiliations:** 1 Faculty of Nursing, University of Alberta, ECHA, 11405-87 Avenue, Edmonton, Alberta T6G 1C9 Canada; 2 School of Nursing, McGill University, Montreal, Quebec Canada; 3 School of Nursing, Dalhousie University, Halifax, Nova Scotia Canada; 4 Canadian Federation of Nurses Unions, Ottawa, Ontario Canada; 5 University of Ottawa, Ottawa, Ontario Canada

**Keywords:** Return to work, Leaves of absence, Mental health issues, Policies, Programs, Practices, Nurses

## Abstract

**Background:**

The complexity of nursing practice increases the risk of nurses suffering from mental health issues, such as substance use disorders, anxiety, burnout, depression, and posttraumatic stress disorder (PTSD). These mental health issues can potentially lead to nurses taking leaves of absence and may require accommodations for their return to work. The purpose of this review was to map key themes in the peer-reviewed literature about accommodations for nurses’ return to work following leaves of absence for mental health issues.

**Methods:**

A six-step methodological framework for scoping reviews was used to summarize the amount, types, sources, and distribution of the literature. The academic literature was searched through nine electronic databases. Electronic charts were used to extract code and collate the data. Findings were derived inductively and summarized thematically and numerically.

**Results:**

Academic literature is scarce regarding interventions for nurses’ return to work following leaves of absence for mental health issues, and most focused on substance use concerns. Search of the peer-reviewed literature yielded only six records. The records were primarily quantitative studies (*n* = 4, 68%), published between 1997 and 2018, and originated in the United States (*n* = 6, 100%). The qualitative thematic findings addressed three major themes: alternative to discipline programs (ADPs), peer support, and return to work policies, procedures, and practices.

**Conclusions:**

While the literature supports alternative to discipline programs as a primary accommodation supporting return to work of nurses, more on the effectiveness of such programs is required. Empirical evidence is necessary to develop, maintain, and refine much needed return to work accommodations for nurses after leaves of absence for mental health issues.

## Introduction

Nursing is considered the largest represented body of workers in healthcare globally [1, 2] with the scope of nursing practice as diverse as it is complex. As nurses engage in their practice and are confronted with the need to address the multiplicity of occupational challenges and demands, their mental health (MH) may be adversely affected [3]. Researchers suggest that psychological challenges such as depression, anxiety, stress, and burnout often result in leaves of absence (LOA) from work for nurses [3–5]. Additionally, these experiences may contribute to the increased incidence of substance use and dependency that results in altered physical health, decreased quality of care, and increased absenteeism [6]. According to the National Council of State Boards of Nursing (NCSBN) [5] “nurses are at greater risk than the overall population for developing problems with substance abuse and addiction” which requires increased attentiveness to the measures in place to reduce, treat, and accommodate nurses with MH issues in the workplace (p. 14).

Considering the risk and factors associated with nurses developing MH issues, we wanted to map the literature about accommodations for nurses returning to work (RTW) after a LOA. As we mapped the literature, we considered practices, policies, and programs as accommodations for nurses’ RTW. In addition to caring for nurses’ holistic health, quality of healthcare delivery, and work absenteeism, this scoping review is significant as we consider the effects of MH on healthcare cost and increased workload for other nurses. Nurse absenteeism is a global concern that is generally the result of health concerns including MH [7]. Mbombi et al. explain that this global concern results in cost of billions of dollars for countries and healthcare organizations. Additionally, these researchers contend that there is a need to address the primary contributing factors that result in LOA and delayed RTW. As a result, there may be decreases in nurses’ workload and burnout, and sustained or improved quality of care provided by nurses attributed to decrease absenteeism.

The aim of this scoping review was to map key themes in the literature about accommodating nurses’ RTW after LOA for MH issues. The objectives were to summarize the amount, types, sources, distribution, and focus of the conceptual and empirical literature. In addition, we identified and discussed key themes and gaps in the literature.

## Methods

This project included a researcher team experienced in scoping review methodology as well as the content areas of policy and nursing workforce. The scoping review methodology provided a systematic, comprehensive, and rigorous mapping of the literature while examining the amount, sources, types, distribution, and focus of the empirical and conceptual literature on a topic [8]. This type of review was also useful for identifying gaps in the literature.

The researchers used a six-stage methodological framework for scoping reviews [8–10] to standardize and clarify the procedures used at each stage of the review. To follow is a description of each stage of the methodological framework and approach used.

### Stage 1

Because a scoping review is intended to summarize a large amount of literature on a topic, research questions for this review were intentionally broad [10]. The review was meant to answer: “What is the scope of the literature about accommodating nurses return to work after leaves of absence for MH issues, including the amount, type, sources, distribution and focus of the conceptual and empirical literature?” and “What are the gaps in the literature?”

### Stage 2

The researchers identified the academic literature and included only primary peer-reviewed literature about accommodating nurses RTW after LOAs for full-text studies available in the English language. No publication date restrictions were imposed. Opinion pieces, press releases, symposia proceedings and letters to the editor, grey literature, and literature specific to registered midwives were excluded.

The researchers in consultation with an experienced research librarian developed the search strategy. The complete literature search strategy can be found in Additional file 1.

### Stage 3

The researchers used a systematic process to select the literature for this scoping review. Records from the search were imported into Endnote, a reference-management program [11], duplicates were identified and discarded, and the remaining records were imported into Covidence, a program specifically created for systematic and scoping reviews [12]. The records were screened in two phases. During phase 1, researchers screened the titles and abstracts of the records to determine their relevance to the review’s purpose and research questions. Records deemed out of the scope of the review were discarded. During phase 2, bibliographic reviews of the remaining papers were completed to locate additional records. The full-text articles were independently assessed to determine if they met the inclusion/exclusion criteria. Articles deemed relevant by one or both of the reviewers were included in the full-text review. Discordant full-text articles were reviewed a second time after which consensus about study eligibility at the full-text review stage was achieved through discussion. Records from full-text screening yielded six records for data extraction and charting. Please find the PRISMA flow diagram [13] used to report the number of records in Additional file 2.

### Stage 4

Researchers extracted and charted the data during this stage. However, to ensure standardization of data extraction and charting across the team [10], SRS developed a charting tool as a Microsoft Word document. The data extraction categories that reflected our research questions were inputted into the Covidence program, and the tool was reviewed and piloted. See the charting tool in Additional file 3.

The researchers used the charting tool to independently extract data from the first five included studies and compared the results [10]. Discrepancies in the coding were discussed among the team, and the tool was refined before proceeding with data extraction. Next, the researchers independently extracted the data from the remaining study. Accurate data collection was ensured with the comparison of each reviewer's independently abstracted data. Discrepancies were discussed to ensure consistency between the reviewers. The data were extracted and compiled into Covidence, a systematic review program, and then downloaded into Microsoft Excel spreadsheets for validation and coding. Standard definitions were developed for each major theme. The minor themes were developed inductively and used to organize the information within each of the major themes.

### Stage 5

Researchers collated the extracted data into numerical and qualitative thematic summaries. To address the research questions, frequencies were used to report the numerical data. The qualitative data were summarized into narrative synthesis. The findings were analyzed in relation to the purpose of the scoping review and the research questions. Gaps in the literature were identified. A quality assessment of the literature was not completed as it was deemed unnecessary for a scoping review [8, 14].

### Stage 6

This stage is considered optional, though Arksey and O’Malley [8] do suggest consulting with key stakeholders can be a useful exercise. However, due to the time and budget constraints for conducting this review, researchers decided to exclude this stage.

The protocol for this review has not been registered.

## Results

Six published records met the inclusion criteria and are discussed in this review. Presented below is (1) types and sources of the literature, followed by (2) numerical and qualitative thematic analysis, and (3) a summary of the gaps in the literature.

### Amount, distribution, sources, and type of evidence

The records (*N* = 6) included in this review are Pan-American, with two from the mid-Atlantic (33%), and one (17%) from the Midwest, north east, north central, and southern regions respectively. Records were published in 1997–2018, with most (*N* = 4, 66%) published between 2015 and 2018, and the remaining records (*N* = 2, 33%) published in 1997–1998. Half (*N* = 3) of the records are journal articles and half (*N* = 3) are thesis dissertations. The majority (*N* = 4, 66%) used quantitative methods, the remaining records (*N* = 2, 33%) used qualitative methods. Each record used a different study design including non-experimental longitudinal, comparative longitudinal, population survey approach, informal telephone survey, grounded theory approach, and phenomenology.

### Numerical thematic analysis

Generally, the literature provided no clear definition for MH issues. However, definitions were provided (*N* = 5, 83%) for terms such as mental disorders, substance, alcohol, and other drugs use as factors contributing to MHI(s). The remaining literature (17%) identified chemical dependence or psychiatric illness affecting cognitive interpersonal or psychomotor health as the defined focus issues of mental health. The population focus for all of the literature were nurses, primarily registered nurses [RNs] and licensed practical nurses [LPNs] (*N* = 3, 50%), only RNs (*N* = 2, 33%) and one record (17%) did not specify the training level of nurses. Half (*N* = 3) of the records did not report a place of employment for nurses, and the remaining half (*N* = 3) reported mixed work settings including but not limited to acute care, specialty areas, community, home care, nurse education, administration non-hospital positions, and long-term care.

### Qualitative thematic findings

These findings were summarized according to three major themes: alternative to discipline programs (ADPs), peer support, and RTW policies, procedures, and practices. Gaps in the literature are presented at the end of each of the themes.

#### Alternative to discipline programs

ADPs primarily focus on nurses with substance use disorder and may be referred to by various terms such as intervention, monitoring, or alternative programs [15–17]. These programs are voluntary, non-punitive programs developed and facilitated by a state board of nursing as an alternative to the traditional disciplinary approach. The literature clearly supports the need for such programs and either directly or indirectly discuss benefits to the nurse and by extension the wider community [17]. Additionally, the literature espouses the humane and rehabilitative approach of ADPs compared to that of the traditional punitive approach [16]. These programs are designed to include after care monitoring of nurses as part of the required steps of program completion, and accommodates the RTW of such nurses primarily through the use of contracts and conferences [18]. The RTW contracts may include various restrictions, e.g., hours of work and administration of narcotics, and the conferences are conducted primarily by nurse managers/supervisors who are responsible for oversight of the nurse upon their RTW [19]. The ADP approach preserves the careers of nurses, by returning much needed nursing personnel back to either previous jobs or other employment [18].

The majority (*N* = 5, 83%) of the literature focused on ADPs addressing the combination of alcohol and other substance use disorders. Only one study (17%) included other MH issues (e.g., psychiatric illness) in addition to alcohol and other substance use disorders. While the benefits of ADPs are directly or indirectly addressed in the literature (*N* = 4, 68 %), little empirical evidence in published peer-reviewed literature addressed the effectiveness of ADPs, the experiences of nurses who participated in ADPs, or a comparison of the experiences of nurses enrolled in ADPs with those involved in traditional disciplinary programs (*N* = 1, 17%). The literature offered no comparison of ADPs of varying lengths and components; neither did the literature offer a gendered approach to explore the experiences of male versus female nurses who have participated in ADPs. Finally, a more in-depth examination is needed of the experiences that nurses with MHIs have with state boards of nursing and ADPs and the impact these experiences have on the RTW process.

#### Peer support

Peer support groups are usually a component of some of the state boards of nursing ADPs and are designed so that nurses guide nurses with alcohol and substance use disorders through recovery [19]. The literature either directly or indirectly discussed the benefits of peer support of nurses as a RTW accommodation. The literature was clear regarding the need for administrative support for a smoother reentry into the workplace by nurses following LOAs for MH issues. More specifically, half of the literature (*N* = 3) identified the support from nurse managers and other colleagues as a major facilitator to RTW of nurses [16, 17, 20]. The literature suggested that for peer support to improve, stigma associated with MH issues, alcohol, and substance use, in particular, must be addressed [20]. Reducing stigma may be improved through education of staff, specifically nursing colleagues [17].

Anecdotally, peer support is seen as a major facilitator for RTW of nurses following LOAs for MH issues; however, there was a paucity of empirical evidence to support this assertion. One study [15] compared the Subjective Sense of Being Supported (SSS) scores of a control group in one state already participating in mandatory peer support groups, with those of the intervention group in a state where the program was piloted. This study found that support groups did not have a statistically significant impact on the compared SSS scores. The hypothesized outcome that the control group with support groups in place would have higher SSS scores than the intervention group prior to attending the trial of nurse support groups was not supported. However, the researcher did acknowledge various limitations that may have adversely affected the outcome.

#### Return to work policies, programs, and practices

These policies, programs, or practices may be either singly or in concert, institutional, national, and state/provincial guidelines, methods, and routines adopted by organizations addressing nurses RTW following LOA for MH issues. The literature mainly focused on alcohol use and other substance use disorders. The state boards of nursing each determine the policies, programs, and practices governing RTW of nurses following MH-related LOA. The literature (*N* = 6, 100 %) directly and indirectly discusses state boards of nursing who continue to use the traditional punitive or disciplinary approach versus state boards on nursing who choose ADPs.

## Discussion

In this review, we provide a synopsis of the scope and content of the primary peer-reviewed literature about the accommodations for nurses’ RTW after LOAs for MH issues. The literature is scant, equally distributed as journal articles and thesis dissertations, and solely Pan-American in scope. Three major themes emerged from the review including ADPs, peer support, and RTW policies, procedures, and practices.

There is an overall paucity of empirical evidence regarding programs, policies, or practices to support nurses RTW following LOA for broader MH issues beyond alcohol and other substance use disorders, e.g., anxiety, depression, and PTSD. Policies guide implementation of programs, practices, and procedures pertaining to RTW of nurses following MH-related LOA; however, less than half (*N* = 2, 30%) of the literature directly address the impact of policies and programs. The literature also demonstrates the lack of standardization regarding policies, programs, and practices even within the same country (*N* = 6, 100%). Finally, the literature clearly demonstrates a need for (1) the development and dissemination of, and (2) precise descriptions of the policies, programs, and practices, (inclusive of each component/step, implementers, etc.) as only half of the studies (*N* = 3) did so.

The limited empirical literature may be indicative of the lack of information, research interest, knowledge, and understanding about the policies, practices, or programs in place to accommodate nurses RTW following LOA for MH issues. The scarcity of literature on this topic is concerning as the plethora of evidence about MH issues among nurses supports the need to better understand RTW accommodations such as programs, policies, and practices [3–5]. Moreover, when researchers study accommodations for RTW after MH-related LOA, nurses are generally discussed as members of the healthcare team who facilitate and coordinate the RTW for workers [21]. It is important that nurses are viewed as susceptible to the same ailments as the general population to avoid potentially ignoring, undertreating, and/or misrepresenting the nursing population in clinical and research practice. Equally important is to consider the adverse effects of not being attentive to nurses with MH issues and the need to have well formulated, documented and widely disseminated policies, programs, and practices in place to ensure safe disclosure, treatment, and RTW.

This scoping review also highlights the lack of attention to the unique gendered experiences in addressing accommodations for nurses RTW following LOA for MH issues. While it is true that nursing is a feminine profession, male nurses can be found among the nursing ranks serving in various capacities. MH issues have been reported to affect as many as one third of healthcare workers with more than two thirds representing nursing staff [22–24]. While the gendered use of substances is not well documented for practicing nurses, male nurses are reported to be three times more likely than female nurses to be affected [25]. Hence, an explicit gendered approach may provide greater insight into how to best tailor the development and implementation for successful accommodations for male and female nurses RTW following LOA for MH issues.

As we consider the apparent lack of academic interest as a potential explanation for the paucity of primary academic literature about RTW accommodations in the form of programs, policies, and services for nurses after MH-related LOA, we are mindful of funding at the federal, regional, and local levels including the government and private sector or lack thereof as a potential contributing factor. Funding may be an influential factor in conducting research to fuel the demand for policies, programs, and practices to be drafted at the organizational, state, or national levels. In turn, literature may be scant due to the inaccurate documentation or categorization of these accommodations.

In addition to research interest, we contemplated professional disinterest in understanding and creating flexible avenues for nurses’ RTW with tailored accommodations. The professional disinterest may be due to the professional desire to protect the image and perception of the nursing profession. The lack of public attention to these issues in the profession may be seen as adventitious to the reputation of the nursing profession [26]. However, lack of visibility of the issue perpetuates the problem and reduces advocacy initiatives such as accommodation for nurses to return to practice.

### Strengths and limitations

The use of methodologically sound and transparent approach to identify and map the literature is one of the many strengths of this review. Additional strengths include the following: (1) The review was conducted by a team of researchers with expertise in scoping review methodology, the substantive area, and knowledge synthesis. (2) An exhaustive search strategy with numerous electronic bibliographic databases was used to identify the peer-reviewed literature. (3) Consistency in identification and coding of the data through the use of standardized screening and data extraction forms which were pilot tested prior to their use.

This review offers a comprehensive examination of peer-reviewed literature addressing accommodations for nurses’ RTW after LOAs for MH issues. A key limitation relates to the body and the nature of the evidence: Only six studies, a small number, were included in the review. Additionally, all but one of the studies employed a cross-sectional design, which precludes drawing conclusions regarding causality between accommodations (i.e., policies, programs and practices) and nurses’ RTW after LOAs for MH issues.

Other limitations include (1) omission of a quality assessment of the studies, since it was deemed unnecessary for a scoping review [8] (2) lack of consultation with key stakeholders, due to time and financial constraints, and (3) potential sources of additional data could have been missed due to the exclusion of full-text articles not available in English.

## Conclusion

This review clearly demonstrates the need for more research focused on accommodations for nurses’ RTW following LOA for MH issues. The literature suggests that ADPs are a more humane approach to treatment, recovery, and RTW of nurses experiencing MH issues; however, little is known about the effectiveness of these programs. Further research is needed to evaluate the effectiveness of ADPs and other accommodations for nurses’ RTW after MH-related LOA. Additionally, more research is needed on the experiences of nurses who participate in these programs, their experiences with their regulatory bodies, and the role gender plays. Empirical evidence is imperative to the development, maintenance, and refinement of much needed programs, policies, and practices to address this crucial topic.

## Supplementary information

**Additional file 1.**

**Additional file 2.**

**Additional file 3.**

## Data Availability

All data generated or analyzed during this study are included in this published article [and its supplementary information files.

## Abbreviations

RTW: Return to work
LOA: Leave(s) of absence
MH: Mental health
ADP: Alternative to discipline program
PTSD: Posttraumatic stress disorder
RN: Registered nurse
LPN/LVN: Licensed practical nurse/licensed vocational nurse
