# Supplementary material for: Mapping the peer-reviewed literature on accommodating nurses’ return to work after leaves of absence for mental health issues: a scoping review
Source: Hum Resour Health. 2020 May 19;18:36. doi: 10.1186/s12960-020-00478-8 (PMC7236175; doi:10.1186/s12960-020-00478-8)
Supplement: Supplementary file 1 — Additional file 1. [file 12960_2020_478_MOESM1_ESM.docx]

Additional file 1

**Complete search strategy**

**Ovid MEDLINE(R) and Epub Ahead of Print, In-Process & Other Non-Indexed Citations and Daily <1946 to April 24, 2019>**

--------------------------------------------------------------------------------

1 exp Mental Disorders/ or Professional Impairment/ or (mental disorder* or mental health or depression or bipolar or anxiety disorder* or mood disorder* or stress disorder* or burnout or burn out or ptsd or ((substance or drug* or opiate* or opioid*) adj3 abus*) or alcohol* or addict* or (attempt* adj2 suicid*)).ti,ab,kf. or (impair* adj (nurse* or professional*)).ti,ab,kf. (1718464)

2 Return to Work/ or Vocational Rehabilitation/ or (((job or work* or employment or employee* or practice) adj4 (reintegrat* or re-integrat* or re-entry or reentry or reenter or re-enter* or return*)) or duty to accommodate).ti,ab,kf. or sick leave/ or (leave of absence or sick leave or sickness absence or illness absence or (leave adj3 (medical or rehabilitat* or recover*))).ti,ab,kf. (30353)

3 1 and 2 (6967)

4 (nurse* or lpn* or rn* or nurs* assistant*).mp. (1373614)

5 3 and 4 (205)

**Embase <1974 to 2019 April 24>**

--------------------------------------------------------------------------------

1 exp mental disease/ or exp drug dependence/ or exp substance abuse/ or (mental disorder* or mental health or depression or bipolar or anxiety disorder* or mood disorder* or stress disorder* or burnout or burn out or ptsd or ((substance or drug* or opiate* or opioid*) adj3 abus*) or alcohol* or addict* or (attempt* adj2 suicid*)).ti,ab,kw. (2582264)

2 return to work/ or vocational rehabilitation/ or exp work resumption/ or (((job or work* or employment or employee* or practice) adj4 (reintegrat* or re-integrat* or re-entry or reentry or reenter or re-enter* or return*)) or duty to accommodate).ti,ab,kw. or (leave of absence or sick leave or sickness absence or illness absence or (leave adj3 (medical or rehabilitat* or recover*))).ti,ab,kw. (34916)

3 1 and 2 (8054)

4 (nurse* or lpn* or rn* or nurse* assistant*).mp. (1769291)

5 3 and 4 (290)

**PsycINFO <1806 to April Week 4 2019>**

--------------------------------------------------------------------------------

1 exp mental disorders/ or impaired professionals/ or exp addiction/ or exp drug addiction/ or intravenous drug usage/ or (mental disorder* or mental health or depression or bipolar or anxiety disorder* or mood disorder* or stress disorder* or burnout or burn out or ptsd or ((substance or drug* or opiate* or opioid*) adj3 abus*) or alcohol* or addict* or (attempt* adj2 suicid*)).ti,ab,id. (975345)

2 reemployment/ or vocational rehabilitation/ or (((job or work* or employment or employee* or practice) adj4 (reintegrat* or re-integrat* or re-entry or reentry or reenter or re-enter* or return*)) or duty to accommodate).ti,ab,id. or (leave of absence or sick leave or sickness absence or illness absence or (leave adj3 (medical or rehabilitat* or recover*))).ti,ab,id. (12247)

3 1 and 2 (3317)

4 (nurse* or lpn* or rn* or nurse* assistant*).mp. (77369)

5 3 and 4 (86)

**CINAHL Plus with Full-text (137 items retrieved)**

(MH "Mental Disorders+") OR "mental disorder*" or "mental health " or depression or bipolar or "anxiety disorder*" or "mood disorder*" or "stress disorder*" or burnout or "burn out" or ptsd or ((substance or drug* or opiate* or opioid*) n3 abus*) or alcohol* or (attempt* n2 suicid*)

AND

(MH "rehabilitation, vocational") or (MH "sick leave") or (job or work* or employment* or employee* or practice) n3 (reintegrat* or "re-integrat*" or "re-entry or reentry or reenter or "re-enter*" or return*) or "duty to accommodate" or "leave of absence" or "sick leave" or "sickness absence" or "illness absence" or (leave n3 (medical or rehabilitat* or recover*))

AND

nurs* or lpn* or rn* or "nurs* assistant*"

**Cochrane Library (Advanced Search/Search Manager; 37 items retrieved)**

#1 [MH "Mental Disorders"] or [mh "Professional Impairment"] or ("mental disorder*" or "mental health" or depression or bipolar or "anxiety disorder*" or "mood disorder*" or "stress disorder*" or burnout or "burn out" or ptsd or ((substance or drug* or opiate* or opioid*) near/3 abus*) or alcohol* or addict* or (attempt* near/2 suicid*)):ti,ab,kw

#2 [mh "Return to Work"] or [mh "rehabilitation, vocational"] or [mh "sick leave"] or (((job or work* or employment* or employee* or practice) near/4 (reintegrat* or "re-integrat*" or "re-entry" or reentry or reenter or "re-enter*" or return*)) or "duty to accommodate" or "leave of absence" or "sick leave" or "sickness absence" or "illness absence" or (leave near/3 (medical or rehabilitat* or recover*))):ti,ab,kw

#3 #1 and #2

#4 (nurse* or lpn* or rn* or "nurs* assistant*"):ti,ab,kw

#5 #3 AND #4

**Scopus (Advanced Search; 166 items retrieved)**

TITLE-ABS("mental disorder*" or "mental health" or depression or bipolar or "anxiety disorder*" or "mood disorder*" or "stress disorder*" or burnout or "burn out" or ptsd or ((substance or drug* or opiate* or opioid*) w/3 abus*) or alcohol* or addict* or (attempt* w/2 suicid*)) AND TITLE-ABS(((job or work* or employment* or employee* or practice) w/4 (reintegrat* or "re-integrat*" or "re-entry" or reentry or reenter or "re-enter*" or return*)) or "duty to accommodate" or "leave of absence" or "sick leave" or "sickness absence" or "illness absence" or (leave w/3 (medical or rehabilitat* or recover*))) AND TITLE-ABS(nurse* or lpn* or rn* or "nurs* assistant*")

**Web of Science Core Collection (Advanced Search; 228 items retrieved)**

TS=("mental disorder*" or "mental health" or depression or bipolar or "anxiety disorder*" or "mood disorder*" or "stress disorder*" or burnout or "burn out" or ptsd or ((substance or drug* or opiate* or opioid*) near/3 abus*) or alcohol* or addict* or (attempt* near/2 suicid*)) AND TS=(((job or work* or employment* or employee* or practice) near/4 (reintegrat* or "re-integrat*" or "re-entry" or reentry or reenter or "re-enter*" or return*)) or "duty to accommodate" or "leave of absence" or "sick leave" or "sickness absence" or "illness absence" or (leave near/3 (medical or rehabilitat* or recover*))) AND TS=(nurse* or lpn* or rn or rns or "nurs* assistant*")

**ProQuest Dissertations & Theses Global (35 items retrieved)**

"mental disorder*" or "mental health" or depression or bipolar or "anxiety disorder*" or "mood disorder*" or "stress disorder*" or burnout or "burn out" or ptsd or ((substance or drug* or opiate* or opioid*) near/3 abus*) or alcohol* or addict* or (attempt* near/2 suicid*)

AND

((job or work* or employment* or employee* or practice) near/4 (reintegrat* or "re-integrat*" or "re-entry" or reentry or reenter or "re-enter*" or return*)) or "duty to accommodate" or "leave of absence" or "sick leave" or "sickness absence" or "illness absence" or (leave near/3 (medical or rehabilitat* or recover*))

AND

nurse* or lpn* or rn or rns or "nurs* assistant*"

**ProQuest ABI Inform (187 items retrieved)**

"mental disorder*" or "mental health" or depression or bipolar or "anxiety disorder*" or "mood disorder*" or "stress disorder*" or burnout or "burn out" or ptsd or ((substance or drug* or opiate* or opioid*) near/3 abus*) or alcohol* or addict* or (attempt* near/2 suicid*)

AND

((job or work* or employment* or employee* or practice) near/4 (reintegrat* or "re-integrat*" or "re-entry" or reentry or reenter or "re-enter*" or return*)) or "duty to accommodate" or "leave of absence" or "sick leave" or "sickness absence" or "illness absence" or (leave near/3 (medical or rehabilitat* or recover*))

AND

nurse* or lpn* or rn or rns or "nurs* assistant*"

**EBSCO Business Source Complete (22 items retrieved)**

"mental disorder*" or "mental health " or depression or bipolar or "anxiety disorder*" or "mood disorder*" or "stress disorder*" or burnout or "burn out" or ptsd or ((substance or drug* or opiate* or opioid*) n3 abus*) or alcohol* or (attempt* n2 suicid*)

AND

(MH "rehabilitation, vocational") or (MH "sick leave") or (job or work* or employment* or employee* or practice) n3 (reintegrat* or "re-integrat*" or "re-entry or reentry or reenter or "re-enter*" or return*) or "duty to accommodate" or "leave of absence" or "sick leave" or "sickness absence" or "illness absence" or (leave n3 (medical or rehabilitat* or recover*))

AND

nurs* or lpn* or rn* or "nurs* assistant*"
