# Supplementary figures and images for: Mapping the peer-reviewed literature on accommodating nurses’ return to work after leaves of absence for mental health issues: a scoping review
Source: Hum Resour Health. 2020 May 19;18:36. doi: 10.1186/s12960-020-00478-8 (PMC7236175; doi:10.1186/s12960-020-00478-8)

Additional File 2 PRISMA Diagram


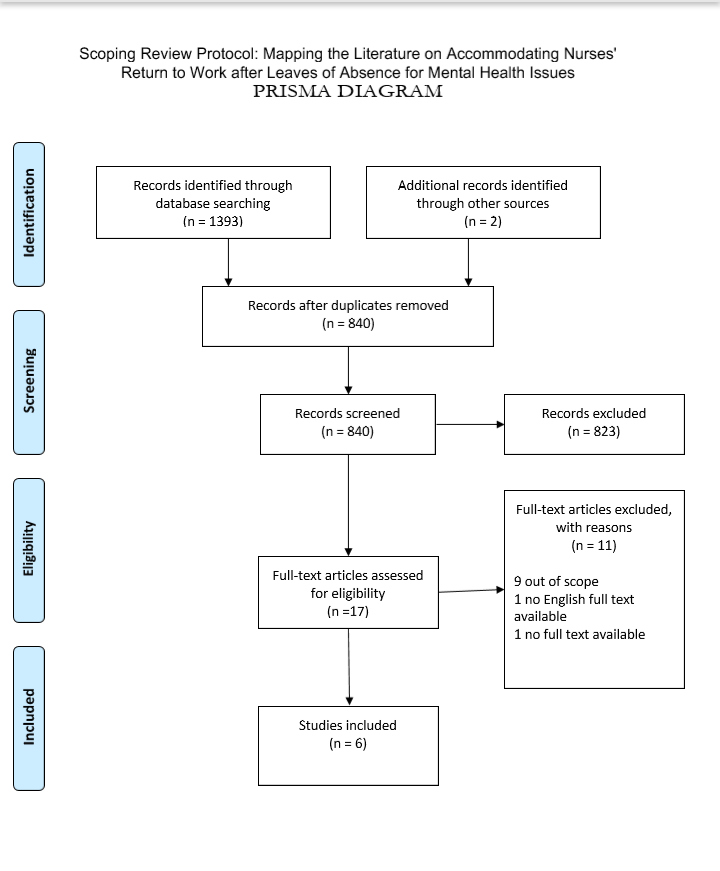

Supplement: Supplementary file 2 — Additional file 2. [file 12960_2020_478_MOESM2_ESM.docx]
