# Supplementary material for: Mapping the peer-reviewed literature on accommodating nurses’ return to work after leaves of absence for mental health issues: a scoping review
Source: Hum Resour Health. 2020 May 19;18:36. doi: 10.1186/s12960-020-00478-8 (PMC7236175; doi:10.1186/s12960-020-00478-8)
Supplement: Supplementary file 3 — Additional file 3. [file 12960_2020_478_MOESM3_ESM.docx]

Additional file 3 Data extraction framework

| **Main Category** | | **Sub-category** | **Description** |
| --- | --- | --- | --- |
| **1.** | Author(s) |  |  |
| **2.** | Title |  |  |
| **3.** | Year of publication |  |  |
| **4.** | Country where the study was conducted (If in Canada, add province or territory) |  |  |
| **5.** | Study methodology |  | Specify whether the study used qualitative, quantitative, or mixed-methods |
| 6. | Description of RTW intervention | Type of intervention        Delivery of intervention      Components of intervention  Length of intervention | Specify the type of intervention of which the study focuses (e.g. program, policy, practice)    Describe how and by whom the intervention is delivered    Describe each part or element of the intervention  Describe for how long the intervention is delivered |
| **7.** | Type of MHIs | Definition of the MHI | Describe the conceptual and operational definitions, and measures of the MHI(s) in the study |
| 8. | Description of the study population | Place of employment            Level of training | Specify if the study targets nurses employed in the public or private sector; acute or long-term, community or home care    Specify if the study targets nurses trained as Registered nurses, registered psychiatric nurses or licensed practical nurses or any combination |
| **9.** | Reported outcomes |  | Describe the intervention outcomes reported in the study |
| **10.** | Effectiveness |  | Describe the results of the intervention testing reported in the study, if applicable |
| **11.** | Barriers |  | Describe the factors that inhibit the implementation of the intervention reported in the study |
| **12.** | Facilitators |  | Describe the factors that support or enable the  intervention of the accommodation reported in the study |
